# A Retrospective Case-Control Study of Eravacycline for the Treatment of Carbapenem-Resistant Acinetobacter Infections in Patients With Burn Injuries

**DOI:** 10.1093/jbcr/irad183

**Published:** 2023-11-16

**Authors:** Connor Alexander, David Hill

**Affiliations:** Department of Pharmacy, Regional One Health, Memphis, TN 38103, USA; Department of Pharmacy, Regional One Health, Memphis, TN 38103, USA

**Keywords:** *Acinetobacter*, burns, CRAB, colistimethate, eravacycline

## Abstract

Thermal injuries lead to a deficiency in one’s natural, protective barrier, resulting in increased susceptibility to pathogens, and often require multiple courses of broad-spectrum antibiotics. Eravacycline (ERA) has shown adequate in vitro activity against multiple multi-drug resistant (MDR) pathogens including *Acinetobacter sp*. Due to the increasing prevalence of MDR bacteria and the heightened susceptibility of patients with burns to infection, studies are needed to examine the clinical effect of eravacycline in this population. The objective of this retrospective, case-control study was to compare the outcomes of patients with thermal injuries treated with eravacycline versus a matched control for carbapenem-resistant *Acinetobacter baumannii* (CRAB) infections. Patients with thermal injury admitted to an American Burn Associated-verified burn center from May 1, 2019 to July 31, 2022, who received eravacycline, were randomly matched 4:1 to a historical cohort using a previously established, de-identified dataset of patients treated with colistimethate sodium (CMS) in the same burn center (March 1, 2009 to March 31, 2014), based on % total body surface area (%TBSA), age, and CRAB. A composite favorable outcome was defined as 30-day survival, completion of the course, lack of 14-day recurrence, and lack of acute kidney injury (AKI). Treatment with eravacycline over CMS was not more or less likely to be associated with a favorable response [odds ratio (95% confidence interval), 2.066 (0.456–9.361), *P* = .347]. Patients treated with CMS had nearly 9-fold higher odds of new-onset AKI versus ERA [8.816 (0.911–85.308), *P* = .06]. Adverse events were uncommon in the ERA group. There was no difference in mortality.

## INTRODUCTION

The emergence of antimicrobial-resistant bacteria is an urgent threat to public health worldwide.^1^ Carbapenem-resistant *Acinetobacter baumannii* (CRAB), specifically, poses a challenge for healthcare providers due to its ability to survive in a hospital setting and persistence on surfaces for extended periods of time.^2^ Critically ill patients residing in an intensive care unit are at a particularly higher risk of being colonized with *A. baumannii*.^3^ The increased use of carbapenems has led to an increased prevalence of carbapenem-resistant bacteria.^4^ Multi-drug resistant (MDR) pathogens pose a threat to public health due to decreased therapeutic options, increased rate of mortality, and increased hospital length of stay.^2,5^

The Centers for Disease Control and Prevention classifies CRAB as an urgent threat due to the limited availability of therapeutic options.^6^ There is currently no consensus guideline regarding the management of CRAB; however, the Infectious Disease Society of America provides expert recommendations on the subject.^7^ The current recommendations include dual therapy with at least 2 agents for moderate to severe infections, with high-dose ampicillin-sulbactam being one of the components. Currently, eravacycline is not recommended due to a lack of clinical data examining its use in CRAB.

Approved by the FDA in 2018 for the treatment of complicated intra-abdominal infections (CIAI), eravacycline is a novel flourocycline that exhibits potent in vitro activity against Gram-positive and Gram-negative bacteria strains resistant to common tetracyclines.^8^ While tetracyclines typically exhibit bacteriostatic activity, ERA has also exhibited bactericidal activity against strains of *A. baumannii*, *Escherichia coli*, and *Klebsiella pneumonia*.^9^ Eravacycline has shown adequate in vitro activity against multiple MDR pathogens including *A. baumannii*.^10^ Eravacycline was determined to be non-inferior to ertapenem and meropenem in regards to the clinical cure rate for CIAIs.^11,12^ The most common adverse effects of ERA are nausea and vomiting.^13^ Advantages of eravacycline include a negligible incidence of acute kidney injury (AKI), as well as no renal dose adjustments. However, ERA has limited activity against *Pseudomonas aeruginosa*.^14^ Mortality rates for CRAB infections have been reported to be as high as 40%-60%; however, a recent multi-center, retrospective, and observational study reported a 30-day mortality rate of 21.9% in patients with CRAB treated with eravacycline.^15,16^

There is currently a gap in the literature regarding the use of eravacycline in the population of patients with burns. Extensive thermal injuries frequently lead to an increased length of hospital stay due to the time required to regrow healthy dermal tissue and eventual wound closure.^17^ Thermal injuries lead to a deficiency in one’s natural, protective barrier to infection, increased susceptibility to multiple pathogens, and often require multiple courses of antibiotics.^18^ For patients surviving the initial 72 h post-burn injury, the most common cause of death is sepsis.^18^ Due to the increasing prevalence of MDR bacteria and the heightened susceptibility of patients with burns to infection, studies are needed to examine the clinical effect of eravacycline in this population. The primary objective of this retrospective, case-control study is to compare the outcomes of patients with thermal injuries treated with eravacycline versus a matched control for CRAB infections.

## METHODS

### Study design and patient population

The study was a retrospective, matched, and case-control study of patients with thermal or inhalation injury admitted to an American Burn Associated-verified burn center from May 1, 2019 to July 31, 2022. Patients admitted during this timeframe, who were at least 18 years of age, had a thermal injury, and were started on eravacycline were eligible for inclusion. Patients were excluded if pregnant, incarcerated, or did not have a CRAB infection. The study period was chosen based on the suspected first use of eravacycline within the burn center. At this time, eravacycline was available via a non-formulary pathway within the center reserved for patients with limited antimicrobial options, based on pathogen sensitivities and recent exposure. Due to the low usage of eravacylcine, patients were randomly matched 4:1 to a historical cohort using a previously established, de-identified dataset of patients treated with colistimethate sodium (CMS) in the same burn center (March 1, 2009 to March 31, 2014), based on % total body surface area (%TBSA), age, and CRAB infection. CMS was the center’s antimicrobial of choice for patients with CRAB infections during the historical period due to limited options for extensively drug-resistant infections. The study received dual Institutional Review Board approval through the hospital and the affiliated university.

### Data collection

Patients were initially screened using admission logs and a database query of all patients started on eravacycline during the study period. The inclusion and exclusion criteria were applied to the initial queries and electronic health records to determine the final study population. Demographic data and treatment characteristics collected included age, sex, race, body mass index (BMI), comorbidities, thermal and inhalation injury, %TBSA, % full thickness, source of infection, total length of stay, mortality, days to mortality, Kirby–Bauer (KB) zone of inhibition (where available), culture results (specimen, pathogens, sensitivities, and resistances), other antimicrobial therapy, length of stay prior to beginning treatment with ERA or CMS, duration of therapy, treatment discontinuation and reason, and adverse drug reactions. The primary source of infection was determined by chart review and the combination of prescriber documentation of infection source and culture results.

Adverse drug reactions were defined as infusion reactions, nausea, vomiting, unexplained acidosis, necrosis of the pancreas, pancreatitis, anaphylaxis, hypersensitivity reactions, azotemia, and other adverse events not previously listed. Treatment success, ERA and CMS dosing and frequency, any dosing regimen changes, if resistance occurred (time, subsequent KB zone size), and AKI before and during therapy [defined as increase in serum creatinine (SCr) by ≥0.3 mg/dL (≥26.5 μmol/L) within 48 h, or increase in SCr to ≥1.5 times baseline; or urine volume < 0.5 mL/kg/h for 6 h] was also collected. Clinical response (ie, treatment success) was defined as 30-day survival, completion of the course (ie, did not change antibiotics due to clinical worsening), and lack of recurrence within 14 days of the final dose of the initial regimen. A composite favorable outcome was defined as 30-day survival, completion of the course, lack of 14-day recurrence, and lack of AKI.

### Statistical analysis

During a priori study planning, the authors anecdotally predicted that 30-40 cases of ERA use could likely be included during the study period. A superiority design was not feasible due to resources being limited to a single-center design. For example, 110 patients (55 per group) would have been required to meet the power of 80%, α of .05, with a predicted 50% success in the control group and 75% success in the experimental group. A non-inferiority study with a 20% difference limit was feasible (*n* = 34 total patients) and could have provided clinically meaningful and novel insight. Due to the low ERA utilization, a 4:1 match to the control group was utilized for outcome analysis via multiple logistic regression. Groups were randomly matched according to age, %TBSA, and presence of CRAB.

Demographic data and treatment characteristics were compared according to outcome, as recommended by Strengthening the Reporting of Observational Studies in Epidemiology guidance.^19^ Dichotomous data were compared via Fisher’s exact test. Continuous data were plotted for visual inspection of normality and tested via the Shapiro–Wilk test. Parametric and non-parametric data were compared utilizing *t*-test or Mann–Whitney *U* test, respectively. A *P* value < .05 for comparisons was considered significantly different. Logistic regression was used to analyze composite favorable outcomes separately. Due to known associations with poor outcomes, it was determined a priori to include age and %TBSA as covariates. Covariate-adjusted survival probability was computed via Cox regression stratified by treatment group. Sigmaplot 11.2 was utilized for the analysis.

## RESULTS

**Figure 1.**
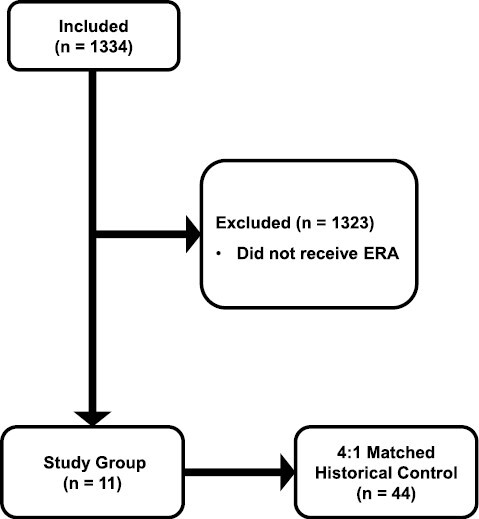
Patient Enrollment. Study Group Was Randomly Matched 4:1 to a Historical Cohort Based on %TBSA Burned, Age, and *A. baumannii* Infection.

**Figure 2.**
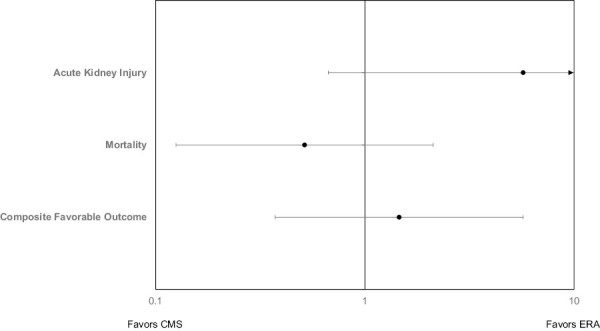
Forest Plot of Outcome According to Treatment Group. Graphically depicted odds ratio (Dot) and 95% confidence interval (Line) or reciprocal, depending on which group is favored. Confidence intervals including the value 1 indicate no difference between groups. For example, patients treated with colistemethate (CMS) over eravacycline (ERA) had a-nearly 9-fold higher likelihood of developing a new acute kidney injury (favors eERA), but did not meet statistical significance in this sample [8.816 (0.911–85.308), *P* = .06]. Of note, the axis is formatted on a log-scale due to the dramatic difference in AKI favoring the eravacycline group. The arrow indicates the line continues off the plotted axis and that of a wide confidence interval. Favorable outcome was defined as 30-day survival, completion of course, lack of 14-day recurrence, and lack of acute kidney injury

A total of 11 patients were treated with eravacycline for a CRAB infection and included in the retrospective study. Patient enrollment is shown in Figure 1. A total of 44 patients were identified from a historical cohort treated with CMS. Patient demographics are listed in Table 1 according to the treatment. There was no difference in age, gender, race, BMI, or %TBSA burned between the treatment groups. There was a significant difference in the primary source of infection between the groups. Burn wound was the primary source of infection in 82% of patients in the ERA group compared to 25% in the CMS group. There was a significant difference in concomitant pathogens with 91% of patients in the ERA group, infected with *Enterbacteriales* compared to just 11% in the CMS group (*P* ≤ .001). Also, 47% of patients in the CMS group had a concomitant *Pseudomonas* infection compared to 0 patients in the ERA group (*P* = .004). The mechanism of thermal injury was flame for all the patients with ERA. The injury mechanism was not captured in the historical dataset. There were no differences in outcomes between patients treated with ERA and CMS (Figure 2).

**Table 1.** Patient Demographics

	Eravacycline (*n* = 11)	Colistimethate (*n* = 44)	*P* value
Age, years^a^	51.3 ± 14.4	48.3 ± 17	.593
Male^b^	10 (90.9)	33 (75)	.422
Caucasian^b^	4 (36.4)	26 (59.1)	.310
BMI, kg/m^2^^c^	29 (23, 34)	27.3 (21.6, 30.7)	.288
TBSA, %^c^	40 (24, 44)	28.6 (17.9, 42)	.293
Primary infection source^b^			.006
Wound	9 (81.8)	11 (25)	
Bacteremia	1 (9.1)	14 (31.8)	
Pneumonia	1 (9.1)	13 (29.6)	
Other	0	6 (13.6)	
Concurrent pathogen^b^			
* Enterbacteriales sp.*	10 (90.9)	5 (11.4)	<0.001
* Pseudomonas sp.*	0	21 (47.7)	0.004

^a^Mean ± standard deviation.

^b^ *n* (%).

^c^Median (interquartile range).

Abbreviations: BMI, body mass index; TBSA, total body surface area.

**Figure 3.**
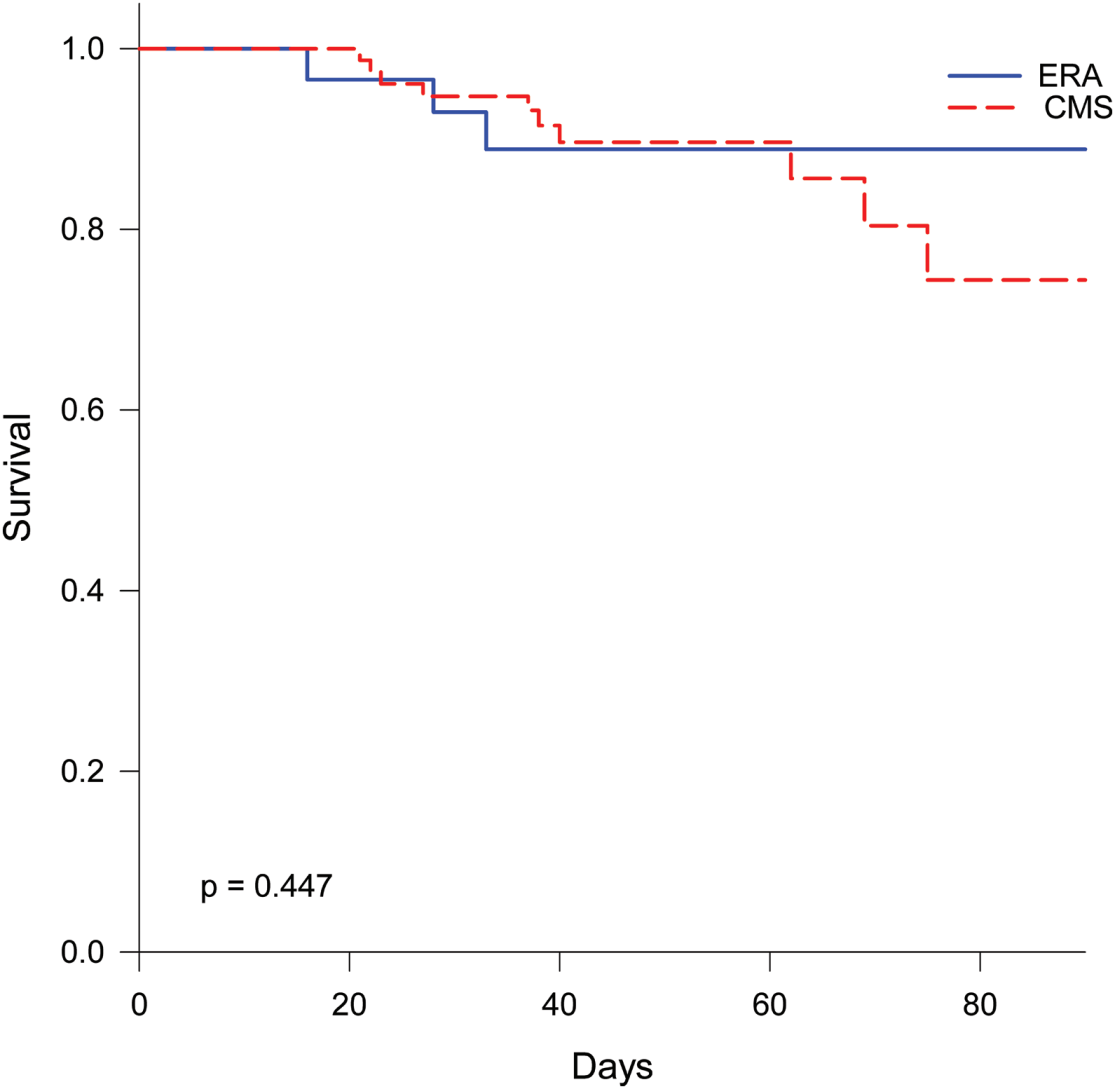
Survival Plot According to Treatment Group. Cox Regression was utilized with age and %TBSA burned as covariates to determine a difference between eravacycline (ERA) and colistimethate sodium (CMS)-treated groups

A favorable outcome occurred in 64% and 55% of patients treated with ERA and CMS, respectively (*P* = .738). There was no significant difference between outcomes based on the primary source of infection or concurrent pathogen. According to covariate-adjusted multivariable logistic regression, the choice to treat with ERA over CMS was not more or less likely to be associated with a favorable response [odds ratio (95% confidence interval), 2.066 (0.456–9.361), *P* = 0.347] (Table 2). Nor was treatment choice associated with new onset AKI (Table 3), although the resultant values are noteworthy. Patients treated with CMS had a numerically higher incidence (36.4% vs 9.1%, *P* = .143) and nearly 9-fold higher odds of new-onset AKI (no previous AKI, but developed after initiating therapy) versus ERA after covariate adjustment [8.816 (0.911–85.308), *P* = 0.06]. There was no difference in mortality after adjusting for age and %TBSA burned (Figure 3). No patients experienced nausea and/or vomiting in the ERA group and incidence was not reported in the CMS historical cohort.

**Table 2.** Multivariable Logistic Regression for Favorable Outcome

Variable	Odds ratio	95% Confidence interval	*P* value
Age, years^a^	0.956	0.919–0.994	.024
Percent TBSA^a^	0.968	0.932–1.006	.095
ERA instead of CMS	2.066	0.456–9.361	.347

^a^A priori determined covariate.

Abbreviations: CMS, colistimethate sodium; ERA, eravacycline.

**Table 3.** Multivariable Logistic Regression for New Onset Acute Kidney Injury

Variable	Odds ratio	95% Confidence interval	*P* value
Age, years^a^	1.039	0.996–1.083	.074
Percent TBSA^a^	1.034	0.993–1.077	.102
CMS instead of ERA	8.816	0.911–85.308	.060

^a^A priori determined covariate.

Abbreviations: CMS, colistimethate sodium; ERA, eravacycline.

ERA susceptibility was known for 6 of the 11 CRAB isolates; however, 9 of the 11 were known to be sensitive to minocycline. The sensitivity of the CRAB was not known for either of the tetracyclines for the 2 patients. Kirby–Bauer disk diffusion zones for eravacycline against CRAB were measured at an average of 17.3 mm. All but one isolate had a zone measuring ≥ 16 mm (range 15–21 mm). The primary sources of infection were burn wound (*n* = 9, 82%), blood (*n* = 1, 9%), and pneumonia (*n* = 1, 9%) in the ERA group, compared to blood (*n* = 14, 32%), pneumonia (*n* = 12, 28%), burn wound (*n* = 11, 25%), and other (*n* = 6, 15%) in the CMS cohort. A total of 91% of patients in the ERA group had concurrent carbapenem-resistant *Enterobacteriaceae* (CRE) compared to 5 patients (11%) who received CMS. Similarly divergent, 64% of patients in the ERA group and 16% in the CMS group were being treated for a concomitant *Stenotrophomonas maltophilia* infection. None in the ERA group were being treated for *Pseudomonas* infection, compared to 48% in the CMS group. Every patient in the ERA group received 1 mg/kg twice daily. The median dose was 100 mg (100, 115 mg) for a median duration of 10 days (8, 16 days). The average total daily dose of CMS was 322 mg per day for a median duration of 17 days (13, 23 days).

## DISCUSSION

To the authors’ knowledge, this is the first study to evaluate eravacycline use for CRAB infections in the population of patients with burns. The primary source of infection was a burn wound. There was no statistically significant difference in microbiological outcomes or mortality between ERA and CMS use for CRAB. CMS resulted in a higher incidence of AKI; however, this difference was not statistically significant. Adverse effects that contributed specifically to ERA were rare in this cohort. This study provides real-world data on the safety and efficacy of ERA use for CRAB, including polymicrobial MDR infections, in a patient population with an increased risk of developing MDR infections.

Acute kidney injury (AKI) is a significant public health problem that leads to increased morbidity and mortality.^20^ Patients with burns having AKI during initial hospitalization have been shown to have increased pulmonary failure, myocardial infarction, hospital length of stay, costs, mortality, and need for renal replacement therapy (RRT).^21^ RRT requires the placement of an additional central line, which increases the incidence of bloodstream infection by 2- to 3-fold.^22^ While not statistically significant, the numerical difference in AKI for CMS-treated patients was clearly emergent; however, this cohort was underpowered (power = 0.217) to adequately determine the presence of a difference. The one patient who experienced an AKI while receiving eravacycline was also receiving known nephrotoxic agents, amphotericin B and amikacin. Eravacycline has a low risk of causing AKI and is primarily metabolized in the liver by oxidation. Polymyxin B is an alternative option for the treatment of CRAB; however, there is fewer outcome data for its use in this population, and some authors have described similar rates of AKI, as CMS.^23^

Concomitant pathogens were not similar between the 2 treatment groups; however, were not different according to outcome. Of the 10 patients with concomitant CRE, a favorable outcome occurred in 7 (70%). Clinical success rates were comparable to phase III and real-world data.^11,12,24–26^ Unfortunately, ERA does not treat *Pseudomonas sp.*; however, its spectrum of activity is fairly broad, including many frequent concomitantly treated pathogens (eg, CRE and *S. maltophilia* in this cohort). Although adverse events (ie, nausea/vomiting) have been described, unfavorable effects were rarely noted with ERA in this cohort, which is contrary to phase III reports and in line with phase IV data.^11,12,25^ At less than $300/day, ERA affords a safe and cost-effective strategy to reduce the number of antimicrobial agents utilized during some infections with MDR pathogens.

There are several limitations in this study. The study was unable to meet the proper power for a non-inferiority study comparing proportions of patients with a favorable outcome, due to the lower-than-anticipated incidence of CRAB and ERA utilization in the burn center. The lower power is depicted in the wide confidence interval [difference (95% confidence interval), 9% (−22.95% to +40.95%)]. Logistic regression is robust and was sufficiently capable of analyzing the smaller numbers of positive events for the composite study outcomes (eg, favorable outcome); however, with 2 covariates and an independent variable, there were not enough events to have sufficient power to analyze the individual components (ie, new AKI, *n* = 17). As such, there is likely a type II error in analyzing the composite endpoint as individual outcomes. Even though the low usage of ERA and the presence of CRAB were challenging for study purposes, the authors are pleased that the incidence is much lower than a decade prior. Hopefully, the results of this study can be combined with other centers’ data to determine eravacycline success from a multi-center population.^25^ Another limitation of this study is the retrospective, single-center design. With such a design, the data are subject to recall bias and regression analysis is not capable of determining causation, only association. Treatment failure rates were relatively high in both groups due to a liberal definition of treatment failure applied, which may overestimate the incidence. Also, treatment failure may have been influenced by the overall severally injured cohorts, the presence of concomitant pathogens, and the use of ERA for reinfection of MDR pathogens. Eravacycline sensitivities were known for most, but not known for each isolate. Kirby–Bauer disk diffusion was the local method utilized to determine sensitivities for non-panel standard antibiotics. In addition, there is no current ERA breakpoint established for CRAB, so zone size for minocycline is typically used (≥16 mm). Similarly, CMS sensitivities are not tested locally, as disk diffusion results for CMS are not reliable. Lastly, inhalation injury data were not available for the CMS group. Inhalation injury is a known contributor to morbidity and mortality and would have ideally been the third covariate included in the analyses. It is noteworthy that 36% of the ERA had a bronchoscopy-diagnosed inhalation injury. Only 1 of the 4 patients with ERA having a concomitant inhalation injury had a favorable outcome. It is not likely the CMS would have had a significantly higher proportion of patients with an inhalation injury, but it is possible and should be considered. If inhalation injury could have been included, the result wound likely move more in favor of ERA or not at all, due to a high incidence in the ERA group. So, the bias was in favor of the control group. There is currently a lack of literature examining the use of ERA in the population of patients with burns, which poses an area for potential future research.

In conclusion, this study is the first to evaluate ERA outcomes with CRAB infections in patients with thermal and inhalation injuries. When compared to a historical cohort treated with CMS, ERA had similar rates of favorable outcomes. The observed incidence of AKI was numerically 4 times less in the ERA group, but the sample was too small to have a proper chance to meet statistical significance. Eravacycline is a potential option to consider in patients with burn injuries being treated for certain polymicrobial, non-Pseudomonal multi-drug-resistant infections.

## References

[1] Ventola CL. The antibiotic resistance crisis: part 2: management strategies and new agents. P T. 2015;40(5):344–352. 25987823 PMC4422635

[2] Manchanda V , Sanchaita S, Singh N. Multidrug resistant acinetobacter. J Glob Infect Dis. 2010;2(3):291–304. 10.4103/0974-777X.68538 20927292 PMC2946687

[3] Cisneros JM , Rodríguez-Baño J. Nosocomial bacteremia due to *Acinetobacter baumannii*: epidemiology, clinical features and treatment. Clin Microbiol Infect. 2002;8(11):687–693. 10.1046/j.1469-0691.2002.00487.x. 12445005

[4] Doi Y. Treatment options for carbapenem-resistant Gram-negative bacterial infections. Clin Infect Dis. 2019;69(Suppl 7):S565–S575. 31724043 10.1093/cid/ciz830 PMC6853760

[5] Fagon JY , Chastre J, Hance AJ, Montravers P, Novara A, Gibert C. Nosocomial pneumonia in ventilated patients: a cohort study evaluating attributable mortality and hospital stay. Am J Med. 1993;94(3):281–288. 10.1016/0002-9343(93)90060-3 8452152

[6] CDC. 2019. Antibiotic resistance threats in the United States of America 2019. https://www.cdc.gov/drugresistance/pdf/threats-report/2019-ar-threats-report-508.pdf

[7] Tamma PD , Aitken SL, Bonomo RA, Mathers AJ, van Duin D, Clancy CJ. Infectious Diseases Society of America guidance on the treatment of AmpC β-lactamase-producing Enterobacterales, carbapenem-resistant *Acinetobacter baumannii*, and *Stenotrophomonas maltophilia* infections. Clin Infect Dis. 2022;74:2089–2114. 34864936 10.1093/cid/ciab1013

[8] Scott LJ. Eravacycline: a review in complicated intra-abdominal infections [published correction appears in Drugs 2019 Apr 11]. Drugs. 2019;79(3):315–324. 10.1007/s40265-019-01067-3 30783960 PMC6505493

[9] Zhanel GG , Cheung D, Adam H, et al. Review of eravacycline, a novel fluorocycline antibacterial agent. Drugs. 2016;76(5):567–588. 10.1007/s40265-016-0545-8 26863149

[10] Morrissey I , Olesky M, Hawser S, et al. In vitro activity of eravacycline against gram-negative bacilli isolated in clinical laboratories worldwide from 2013 to 2017. Antimicrob Agents Chemother. 2020;64(3):e01699–e01719. 10.1128/AAC.01699-19 31843999 PMC7038303

[11] Solomkin J , Evans D, Slepavicius A, et al. Assessing the efficacy and safety of eravacycline vs ertapenem in complicated intra-abdominal infections in the Investigating Gram-Negative Infections Treated With Eravacycline (IGNITE 1) trial: a randomized clinical trial. JAMA Surg. 2017;152(3):224–232. 10.1001/jamasurg.2016.4237 27851857

[12] Solomkin JS , Gardovskis J, Lawrence K, et al. IGNITE4: results of a phase 3, randomized, multicenter, prospective trial of eravacycline vs meropenem in the treatment of complicated intraabdominal infections. Clin Infect Dis. 2019;69(6):921–929. 10.1093/cid/ciy1029 30561562 PMC6735687

[13] Eravacycline. Lexi-Drugs. Lexicomp; 2022. http://online.lexi.com/. Updated September 29, 2022. Accessed December 27, 2022.

[14] Gorham J , Taccone FS, Hites M. Drug regimens of novel antibiotics in critically ill patients with varying renal functions: a rapid review. Antibiotics (Basel). 2022;11(5):546. 10.3390/antibiotics11050546 35625190 PMC9137536

[15] Alosaimy S , Morrisette T, Lagnf AM, et al. Clinical outcomes of eravacycline in patients treated predominately for carbapenem-resistant *Acinetobacter baumannii*. Microbiol Spectr. 2022;10(5):e0047922. 10.1128/spectrum.00479-22 36190427 PMC9602915

[16] Peleg AY , Seifert H, Paterson DL. *Acinetobacter baumannii*: emergence of a successful pathogen. Clin Microbiol Rev. 2008;21(3):538–582. 10.1128/CMR.00058-07 18625687 PMC2493088

[17] Martin K , Arif F, Sultan-Ali I, Velamuri SR, Hill DM. Analysis of ceftazidime/avibactam use for treating carbapenem-resistant infections in critically ill patients with thermal or inhalation injuries. J Burn Care Res. 2022;43(4):759–765. 10.1093/jbcr/irac038 35416248

[18] Lachiewicz AM , Hauck CG, Weber DJ, Cairns BA, van Duin D. Bacterial infections after burn injuries: impact of multidrug resistance. Clin Infect Dis. 2017;65(12):2130–2136. 10.1093/cid/cix682 29194526 PMC5850038

[19] von Elm E , Altman DG, Egger M, Pocock SJ, Gotzsche PC, Vandenbroucke JP. The Strengthening the Reporting of Observational Studies in Epidemiology (STROBE) statement: guidelines for reporting observational studies. *Lancet*. 2007;370(9596):1453–1457. 10.1016/S0140-6736(07)61602-X 18064739

[20] Singbartl K , Kellum JA. AKI in the ICU: definition. Kidney Int. 2012;81(9):819–825. 10.1038/ki.2011.339 21975865

[21] Thalji SZ , Kothari AN, Kuo PC, Mosier MJ. Acute kidney injury in burn patients: clinically significant over the initial hospitalization and 1 year after injury: an original retrospective cohort study. Ann Surg. 2017;266(2):376–382. 10.1097/SLA.0000000000001979 27611620 PMC5342949

[22] Dhingra RK , Young EW, Hulbert-Shearon TE, Leavey SF, Port FK. Type of vascular access and mortality in US hemodialysis patients. Kidney Int. 2001;60(4):1443–1451. 11576358 10.1046/j.1523-1755.2001.00947.x

[23] Nation RL , Velkov T, Li J. Colistin and polymyxin B: peas in a pod, or chalk and cheese? Clin Infect Dis. 2014;59(1):88–94. 10.1093/cid/ciu213 24700659 PMC4305129

[24] Alosaimy S , Molina KC, Claeys KC, et al. Early experience with eravacycline for complicated infections. Open Forum Infect Dis. 2020;7(5):ofaa071. 10.1093/ofid/ofaa071 32411809 PMC7210802

[25] Alosaimy S , Molina KC, Bouchard J, et al. Real world multi-center experience with eravacycline for complicated infections. 2020;7:ofaa071. 10.1093/ofid/ofaa071. PMC7210802 32411809

[26] Carr AL , Ghali AE, Kaur P, et al. Early real-world evidence in the use of eravacycline for the management of draconian infections. 2020;7(Suppl 1):S651–2. https://doi.org10.1093/ofid/ofaa439.1454.

